# A Patient Specific Biomechanical Analysis of Custom Root Analogue Implant Designs on Alveolar Bone Stress: A Finite Element Study

**DOI:** 10.1155/2016/8242535

**Published:** 2016-05-04

**Authors:** David Anssari Moin, Bassam Hassan, Daniel Wismeijer

**Affiliations:** Department of Oral Function and Restorative Dentistry, Academic Centre for Dentistry Amsterdam (ACTA), Research Institute MOVE, 1081 LA Amsterdam, Netherlands

## Abstract

*Objectives.* The aim of this study was to analyse by means of FEA the influence of 5 custom RAI designs on stress distribution of peri-implant bone and to evaluate the impact on microdisplacement for a specific patient case.* Materials and Methods*. A 3D surface model of a RAI for the upper right canine was constructed from the cone beam computed tomography data of one patient. Subsequently, five (targeted) press-fit design modification FE models with five congruent bone models were designed: “Standard,” “Prism,” “Fins,” “Plug,” and “Bulbs,” respectively. Preprocessor software was applied to mesh the models. Two loads were applied: an oblique force (300 N) and a vertical force (150 N). Analysis was performed to evaluate stress distributions and deformed contact separation at the peri-implant region.* Results*. The lowest von Mises stress levels were numerically observed for the Plug design. The lowest levels of contact separation were measured in the Fins model followed by the Bulbs design.* Conclusions*. Within the limitations of the applied methodology, adding targeted press-fit geometry to the RAI standard design will have a positive effect on stress distribution, lower concentration of bone stress, and will provide a better primary stability for this patient specific case.

## 1. Introduction

With technological advances in the field of implant dentistry novel treatment modalities and more efficient options became available. The custom 3D printed root analogue implant (RAI) as defined by Anssari Moin et al. [1, 2] and Figliuzzi et al. [3] is a futuristic treatment option for immediate implantation and immediate loading cases for a soon to be removed tooth. Advantages of the RAI technique when compared to conventional screw shaped multipiece implants may encompass more cost efficiency, one-piece implant, and minimal traumatic surgical intervention [1–6].

An essential factor for realization of all implant-based prosthetic reconstructions is successful osseointegration of the implant. In particular, primary stability plays a fundamental role in one-stage implant surgery with or without immediate loading [7]. Conventional screw type implants achieve primary stability through mechanical fixation by implant threads in bone [8]. Numerous studies on the factors influencing primary stability (implant shape specifications, surface modifications, bone quality, and surgical technique) and the effect on the process of osseointegration have been performed [8–11]. However, primary stability for the RAI technique is based on the (targeted) press-fit phenomenon for achieving successful osseointegration [1–3, 6]. Since the custom RAI is based on Cone Beam Computed Tomography (CBCT), Computer Aided Design (CAD), and 3D printing technology an unlimited array of designs for this custom implant approach is available. Every RAI design option aimed at increasing initial mechanical stability for the root part of the RAI will have a different biomechanical effect on the surrounding bone and influence on the relative microdisplacement at bone-to-implant interface consequently leading to diverse osseointegration results, bone resorption, or failures.

Finite Element Analysis (FEA) has become an effective method in investigating bone stress/strain around implants and relative microdisplacement between bone-to-implant interfaces [12]. However, as with all FEA studies the analysis is confined to a limited amount of factors and designs and cannot be generalised, specifically not for individual cases. Thus, the aim of this study is to analyse, with the means of FEA, the influence of 5 custom RAI designs on stress distribution of peri-implant bone and to evaluate the impact on microdisplacement for a specific patient case.

## 2. Materials and Methods

### 2.1. Model Design

**Figure 1:**
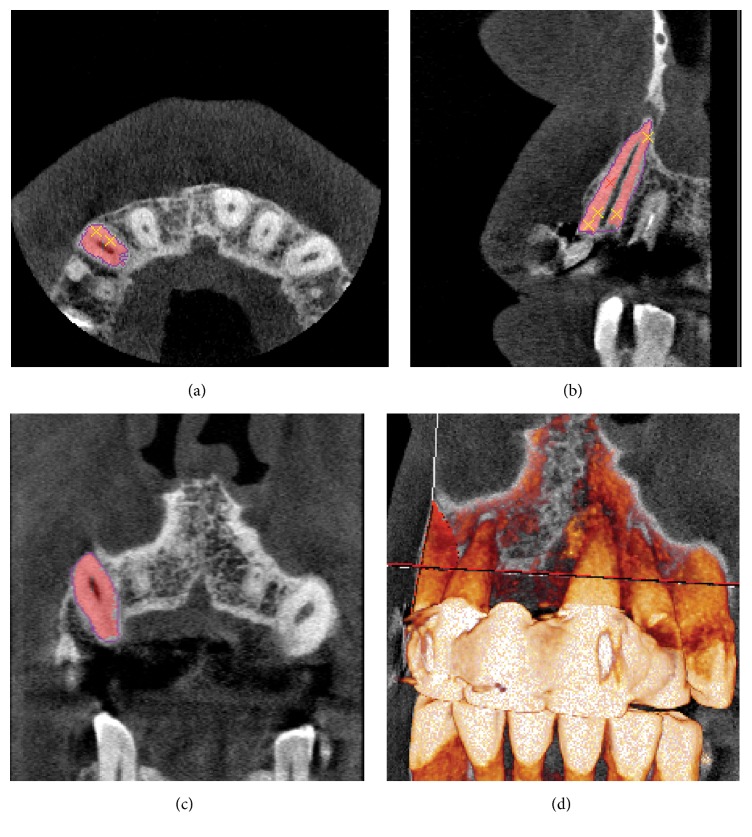
Segmentation and preparation of the RAI. Coronal (a), axial (b), sagittal (c), and 3D (d) views.

A patient (male, 64 years of age) presenting a profoundly decayed upper right canine was selected and informed consent was obtained from the patient. Based on the method previously described by Anssari Moin et al. [1, 2] a 3D surface model of RAI was constructed. In brief the procedure was as follows: the patient was scanned with the 3D Accuitomo 170 CBCT system (Accuitomo 170, 90 kVp, 5 mA, 30.8 s, 4 × 4 cm Field of View [FoV], voxel 0.08 mm^3^, Morita Inc., Kyoto, Japan) using the recommended scan protocol. Amira software (v4.1, Visage Imaging, Carlsbad, CA) was used for image analysis. A region of interest limited to the tooth and its surrounding was initially selected and a threshold segmentation algorithm based on histogram analysis of grey values was used to separate the tooth (root and crown) from surrounding bone and periodontium. Further semiautomated segmentation based on slice-by-slice analysis was implemented to enhance the segmentation by removing any residual artifacts (Figure 1). The segmented dataset was converted to 3D surface model using the marching cube algorithm and saved in the standardized triangulation language (STL) file format.

**Figure 2:**
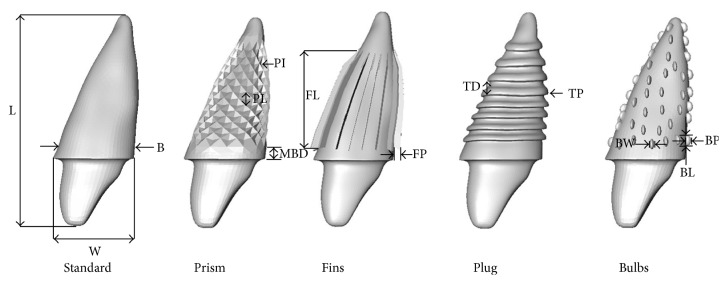
3D models of the 5 designs analyzed. Dimensions and notation of geometric properties are as follows: L: total implant length 26.90 mm similar to all designs, W: maximum width of basic implant body 9.55 mm, MBD: shoulder margin to bone (B) distance 2 mm for all models, PI: Prism maximum intrusion 0.85 mm, PL: Prism maximum length 1.65 mm, FP: Fins protrusion 0.80 mm, FL: Fins length 12.90 mm, TP: thread protrusion 0.30 mm, TD: thread maximum distance 1.50 mm, BP: Bulbs protrusion 0.50 mm, BW: Bulbs width 0.55 mm, and Bl: Bulbs length 1.20 mm.

Based on the STL model five different (targeted) press-fit design RAI FE models have been constructed using 3D CAD software (Inventor*™*, Autodesk GmbH, Munich, Germany). For the five RAI models a Standard identical abutment, based on morphological expectation of the original tooth crown and measurements on neighboring teeth, was designed at 2 mm distance coronal from the expected bone level after implantation. Subsequently, the following (targeted) press-fit design modifications were constructed: (1) nonmodified Standard, (2) targeted press-fit Prism, (3) targeted press-fit Fins, (4) targeted press-fit Plug, and (5) targeted press-fit Bulbs, referred to as “Standard,” “Prism,” “Fins,” “Plug,” and “Bulbs,” respectively.  Figure 2 shows the five designs with description of the different geometrical characteristics.

Five bone models surrounding 3 mm congruent to the respective RAI models were built using Femap software (v. 11.0.1, Siemens PLM Software, Plano, TX, USA).

**Figure 3:**
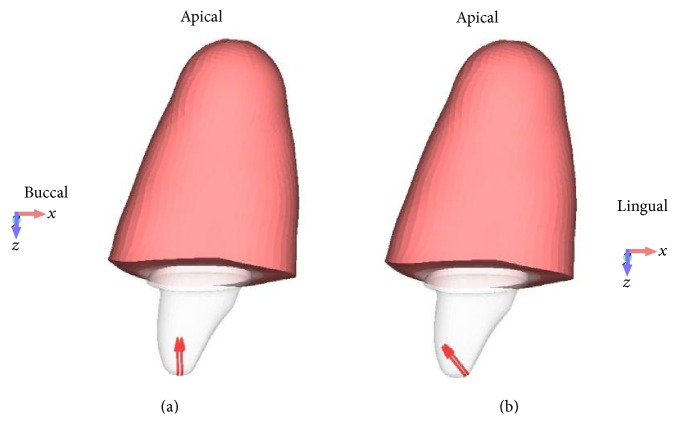
Overall illustration of a meshed model. The red vectors indicating the direction of the applied vertical (a) and oblique (b) forces.

Finally, preprocessor software (Femap v. 11.0.1, Siemens PLM Software, Plano, TX, USA) was applied to mesh the models with quadratic tetrahedral solid elements (Figure 3). Mesh refinement based on convergence analysis resulted in a mesh size of 0.5 mm.  Table 1 summarizes the number of elements and nodes for each model.

### 2.2. Material Properties, Interface, Constrains, and Loading Conditions

The following assumptions were made for the RAI FE models: composition of a titanium alloy Ti6Al4V, Young's modulus *E* = 110 GPa, and Poisson's ratio *ν* = 0.35 with the material being homogeneous, isotropic, and linearly elastic [13, 14].

The bone models were constructed using a homogenous isotropic linearly elastic material of 1 mm inner cortical layer (Young's modulus *E* = 12.6 GPa, Poisson's ratio *ν* = 0.3, and Shear modulus *G* = 5.7 GPa) and a 2 mm outer trabecular layer (Young's modulus *E* = 1.1 GPa, Poisson's ratio *ν* = 0.3, and Shear modulus *G* = 0.07 GPa) as proposed in the reviewed literature [13, 14].

Bone-to-implant interfaces were assumed to be frictional surfaces to represent a nonosseointegrated contact situation. A Coulomb frictional method (coefficient of friction = 0.3) was adopted to define linear contact behavior [14, 15].

Two loads were applied to simulate anterior bite force: an oblique buccoapical force with a magnitude of 300 N set on 135° to the long axis of the implant and a vertical force in apical direction to the long axis of the implant with a magnitude of 150 N, as shown in Figure 3 [16, 17].

The nodes in the outer surrounding layer of trabecular bone were constrained in all directions (zero nodal displacement).

### 2.3. Analysis

Numerical solving (Nastran v. 8.0, Siemens PLM software, Plano, TX) and postprocessor analysis (Femap v. 11.0.1, Siemens PLM software, Plano, TX, USA) was performed on the meshed bone-implant models to evaluate stress distributions on cortical and trabecular bone and deformed contact separation (micromotion) at the peri-implant region.

Based on previous research the following measurements were recorded: the von Mises equivalent stress (*σ*_VM_) at the bone peri-implant interface as a quantity of stress level for the load transfer mechanism [12, 18–21], the tensile/maximum (*σ*_*t*_) and compressive/minimum (*σ*_*c*_) principal stresses as a criterion to evaluate the bone overloading [19, 20], and finally deformed contact separation (micromotion in *μ*m) as an indicator for initial implant stability [22, 23].

## 3. Results

**Figure 4:**
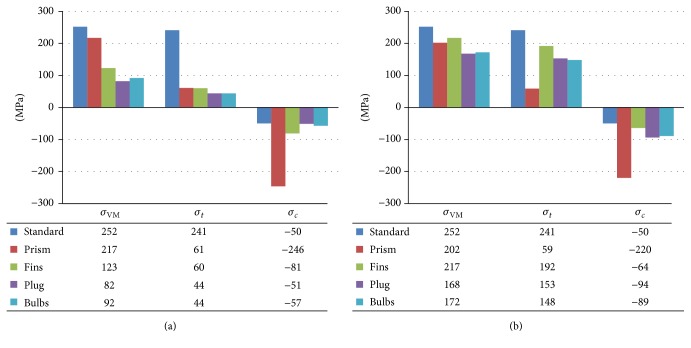
Comparison of the maximum von Mises equivalent stress (*σ*_VM_) and the tensile (*σ*_*t*_) and compressive (*σ*_*c*_) principal stresses under vertical (a) and oblique (b) loading components in the 5 designs.

Figure 4 displays the average measured stress values (in MPa) of the principal and von Mises stresses at the supporting tissues for all groups. Notably, on average the stress levels caused by oblique loading were higher when compared to vertical loading.

The Standard design RAI exhibited the highest von Mises stress and highest minimum principal stress values (highest compressive stress) under both loading conditions (*σ*_VM_ = 252 and *σ*_*c*_ = −50). The lowest von Mises stress levels were numerically observed for the Plug design under the different loading conditions (Figure 4(a), *σ*_VM_ = 82; Figure 4(b), *σ*_VM_ = 168), indicating a reduction of 67.4% and 33.3%, respectively, when compared to the Standard design. Furthermore, the highest measured tensile stress in cancellous bone was 4 MPa for the Standard design (data not shown).

**Figure 5:**
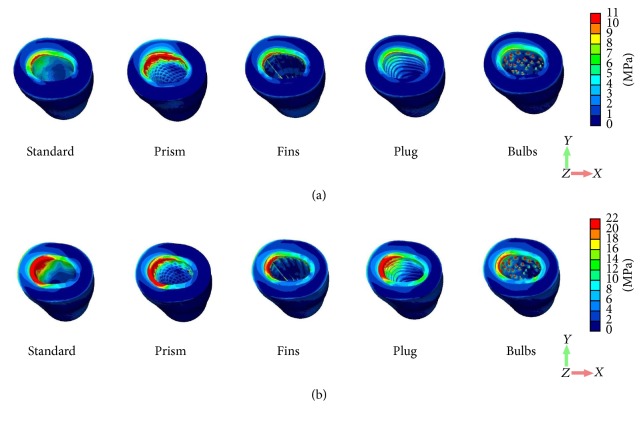
Distribution patterns of von Mises stress under vertical (a) and oblique (b) loading components.

**Figure 6:**
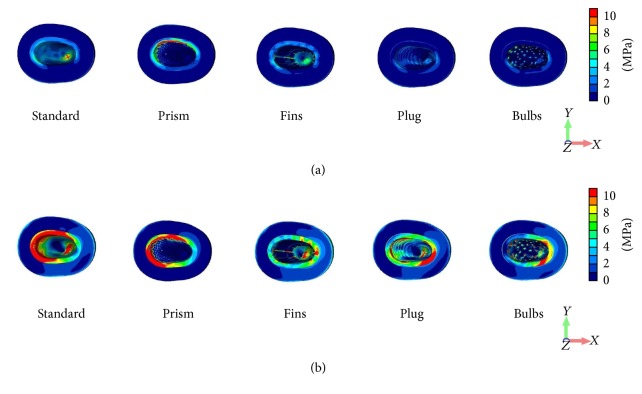
Distribution patterns of the tensile principal stress under vertical (a) and oblique (b) loading components.

Comparing behavior of von Mises stress distribution caused by vertical (Figure 5(a)) and oblique loading (Figure 5(b)), it can be observed that the cortical peri-implant bone exhibited greater stress concentration than trabecular bone. In tension stress, concentrations can be noted at the loaded side for the Standard and Prism under the oblique loading component (Figure 6).

However, under the same conditions the Plug, Fins, and Bulb designs showed tensile stress intensities on the lingual side and in the buccal area of the protrusive extensions of the design (Figure 6).

**Figure 7:**
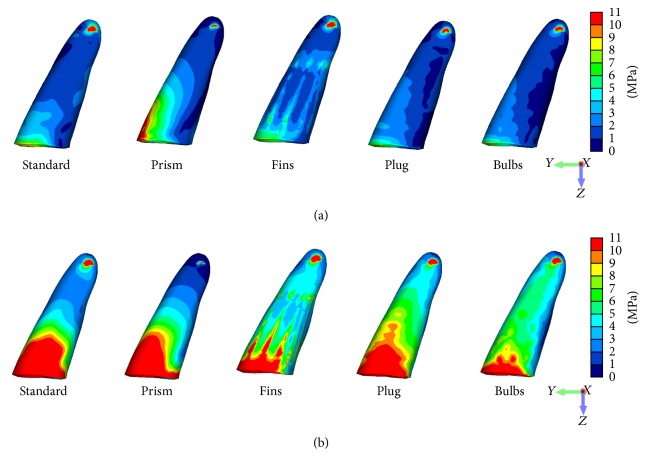
Distribution patterns of von Mises stress in the cortical outer layer of the surrounding bone under vertical (a) and oblique (b) loading components.

**Figure 8:**
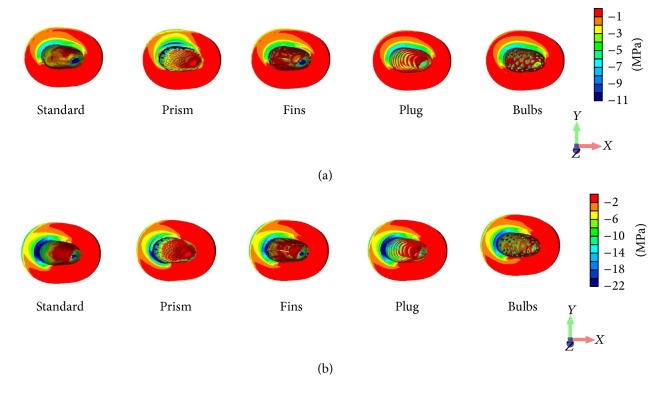
Distribution patterns of the compressive principal stress under vertical (a) and oblique (b) loading components.

The apical peri-implant area indicated high von Mises stress concentrations in all designs (Figure 7) and tensile stress peaks under both loading conditions for the Standard, Plug, and Fin designs. Comparison of the minimum principal stress illustrated in all models the highest compressive stress concentrations on the lingual side (Figure 8).

Table 2 shows the microdisplacement of the various RAI designs from the peri-implant bone with respect to the loading conditions. The highest magnitude of micromotion level was measured in the Prism design, 32.10 *μ*m and 32.51 *μ*m under vertical and oblique loading, respectively. Remarkably, the lowest levels of contact separation were measured in the Fins model followed by the Bulbs design under vertical and oblique forces: 5.45 *μ*m, 6.25 *μ*m and 6.35 *μ*m, 6.42 *μ*m, respectively. Microdisplacement patterns were located at neck area in direction of the forces and in contra lateral direction in the apical area in all designs (images not shown).

## 4. Discussion

In this study five different designs of RAI were analyzed for stress-based biomechanical behavior for a specific patient by means of finite element simulations. In the primary phase of endosseous healing multiple biomechanical mechanical factors play a role. The von Mises stress was used as an indicator for the load transfer mechanism, principal stresses as indicator to bone overloading and micromotion as indicator for initial stability. Numerical results from the current study suggest that adding targeted press-fit design characteristics to the Standard RAI design will decrease the amount of maximum von Mises stress in the surrounding peri-implant bone, subsequently leading to more favorable load behavior for this patient. Previous studies have assumed maximum bone strength as biological limit to bone failure and activation of bone resorption [15, 19, 21]. Correspondingly, it has been proposed that overloading of cortical bone occurs when the maximum compressive principal stress exceeds −190 MPa and maximum tensile principal stress exceeds 130 MPa [15, 19, 21]. Likewise, trabecular bone overloading will occur when the compressive and tensile principal stresses exceed −5 MPa and 5 MPa, respectively [15, 19, 21]. According to the result of this study it has been found that solitary Prism design exceeded the maximum compressive stress criterion for cortical bone. The Standard, Fins, Plug, and Bulbs designs exceeded the tensile stress threshold in cortical bone. The threshold for trabecular bone overloading in tension was not reached. However, when observing the compressive stresses under oblique loading in trabecular bone, it can be noted that in the regions of the implant neck all implant designs exceeded the biological limit, inducing a risk to bone loss (Figure 8). The Fins and Bulbs designs showed the lowest levels of micromotion, indicating the most favorable primary stability. Nonetheless, it must be noted that the influence of micromotion on osseointegration is of scientific debate as some studies have suggested a more positive effect on the tissue differentiation and bone formation around implants under controlled micromotion up to 50 *μ*m [24]. Additionally, in our study it has been found that the higher oblique loading component causes more stress concentrations on cortical and trabecular bone when compared to vertical loading. Therefore, oblique loading in the primary stage after implantation will have a more negative effect on bone healing and should be minimized.

In this current study multiple drawbacks and limitations should be named. The peri-implant surrounding bone was modeled and assumed as a homogeneous, isotropic, linearly elastic material. However, it is known that the biomechanical behavior of this living tissue is heterogeneous, anisotropic, and nonlinear [14, 19, 20]. Moreover, a 100% osseous contact between implant and bone was assumed. Contact relationship between implant and bone was defined as linear contact behavior by using a Coulomb frictional model. Although contact behavior should be defined in a nonlinear method, several studies are in agreement about adopting a linear frictional model since non-linear contact analysis is highly complex [14, 25]. In clinical situations the actual bone-to-implant contact directly after insertion of the RAI will be dependent on many factors, that is, accuracy of the RAI technique on multiple levels, (periapical) bone defects, and surgical handling. The quantity of in situ osseous contact after implantation of the RAI will have profound effect on primary stability and stress behavior. Furthermore, the herein applied loads were static one directional loads of amplitude of 150 N (vertical) and 300 N (oblique) whereas in clinical situations considerably variable loads can be observed depending on the location of the RAI in the oral cavity and patient characteristics. Despite the fact that simulation methods and FE modeling were beyond the scope of this investigation, the current limitations can be considered as acceptable in a numerical sense and are in agreement with multiple studies [13, 14, 16, 17, 19, 25].

Especially with the rise of custom 3D printed implants questions concerning biomechanical behavior in each specific patient surface. Ideally for future implementation of custom 3D designed and printed implants easy accessible individual patients specific FEA should be performed to get a better understanding of the biomechanical behavior of different implant designs for a specific case.

## 5. Conclusion

Based on the results of this study and within the limitations of the applied methodology, it has been found that adding targeted press-fit geometry to the RAI Standard design, preferably Fins or Bulbs, will have a positive effect on stress distribution and lower concentration of bone stress and will provide a better primary stability for this patient case.

## Figures and Tables

**Table 1:** Number of elements and nodes used in the 5 FE models.

Model	Elements	Nodes
Standard	235094	336907
Prism	212965	306454
Fins	211820	309433
Plug	389742	567419
Bulbs	371570	550137

**Table 2:** Micromotion measures (*μ*m) on the various models.

Model	Micromotion (*μ*m) under vertical loading	Micromotion (*μ*m) under oblique loading
Standard	10.90	11.72
Prism	32.10	32.51
Fins	5.45	6.25
Plug	9.88	10.69
Bulbs	6.35	6.42
